# Critical Correlation Between Obesity and Cardiovascular Diseases and Recent Advancements in Obesity

**DOI:** 10.7759/cureus.51681

**Published:** 2024-01-04

**Authors:** Mahesh Pakhare, Ashish Anjankar

**Affiliations:** 1 Medical Education, Jawaharlal Nehru Medical College, Datta Meghe Institute of Higher Education and Research, Wardha, IND; 2 Pathology, Jawaharlal Nehru Medical College, Datta Meghe Institute of Higher Education and Research, Wardha, IND

**Keywords:** dyslipidemia, diabetes, hypertension, bariatric surgery, gut microbiome, liposuction, cardiovascular disease, obesity

## Abstract

Scientific literature has investigated and well-documented the complex relationship between obesity and cardiovascular diseases. Obese people are much more likely to have atrial fibrillation, dyslipidemia, diabetes, and coronary artery disease, among other cardiovascular conditions. Additionally, obesity poses a severe risk for diseases like hypertension, heart failure, and atherosclerotic heart diseases, affecting various aspects relating to their underlying mechanisms, diagnosis, and clinical effects. This article summarizes recent developments in our understanding of and response to obesity. Pharmacotherapy, gut microbiome research, bariatric surgery, digital health solutions, behavioral interventions, and precision medicine are just a few of the fields in which these developments have been made. While liposuction offers a less invasive option for redistributing volume and getting positive results, bariatric surgery remains the most effective treatment for severe obesity. Emphasis is placed on the pathophysiological mechanisms that underlie the complex interactions between obesity and a number of diseases, such as atrial fibrillation, diabetes, hypertension, dyslipidemia, and coronary artery disease. The significance of lifestyle changes in reducing the cardiovascular risks associated with obesity, such as atrial fibrillation and heart disease, is emphasized. To improve overall cardiovascular health and achieve better clinical outcomes, obesity must be promptly identified and actively managed. Investigations into how the gut microbiome affects obesity, the creation of novel pharmacological treatments for appetite suppression and metabolic enhancement, improvements in bariatric surgery methods that emphasize patient success and safety, as well as creative digital health solutions and behavioral treatments, are some examples of emerging research fields. In addition, precision medicine approaches, including the modulation of the gut microbiome through dietary changes and supplements, hold great promise in combating obesity and its associated comorbidities and have the potential to revolutionize the management of obesity by tailoring treatments to the specific needs of individual patients.

## Introduction and background

Obesity and cardiovascular diseases (CVD) do have a strong correlation, which has been well-studied and documented in the scientific literature. The scientific and medical communities generally concur that obesity is one of the most common pressing community health issues of this century. Obesity is becoming more common and a growing concern for global health in both developed and developing countries. According to the World Health Organisation (WHO) statistics, 13% of people over the age of 18 worldwide are obese, and 39% of those are overweight [1]. Numerous cardiovascular diseases, such as dyslipidemia, diabetes, atrial fibrillation, high blood pressure, heart failure, and stroke, are significantly increased by obesity. Over the last few decades, obesity has become a global epidemic. Obesity is associated with a high frequency of impaired glucose tolerance (IGT, sometimes called prediabetes), a different risk factor for diabetes type 2 [2]. 

Body mass index (BMI), a measure of a person's weight status that identifies whether or not they are overweight or obese, is determined by the split of their weight in pounds by their height in square meters. Overweight and obesity are defined by BMI levels over 25 and 30, respectively, and have been linked to a higher risk for acquiring a variety of chronic diseases, including type 2 diabetes, coronary artery disease, and carcinoma [3]. The most extensive and significant study describing the current worldwide incidence of adult and child obesity is undoubtedly already available [2,3]. BMI is an indication of obesity, but it does not provide data on the abundance of fat, which is crucial for determining cardiovascular risk. To help distinguish between central or abdominal obesity, two new clinical measurements measuring waist-to-hip proportion and abdominal circumference were produced. A person's gastrointestinal estimation (waist circumference) must be more than 102 cm for males and 88 cm for females to be regarded as centrally obese. It raises the probability of acquiring cardiovascular disease [1]. Central obesity is indicated by a waist-to-hip ratio of more than 0.85 for women and more than 0.9 for men [4]. The following review article focuses on how obesity affects the pathophysiology, diagnosis, management, and clinical results of heart failure (HF) and atherosclerotic CVD, including arrhythmias, atrial fibrillation (AF), and particularly sudden cardiac death (SCD). Numerous factors include CVD, including stroke, pulmonary hypertension, and venous thromboembolic disease [5].

Several research have recently been published to study obesity and its treatment. Until September 2021, the following notable developments included pharmacotherapy, gut microbiome, bariatric surgery, digital health solutions, behavioral interventions, and precision medicine. In both directions, the relationship between obesity and gut microbiota (GM) has received a lot of attention recently, with obesity being seen both as a result of and a cause of GM disorder [6]. Patients who are extremely obese find it difficult to lose weight and, more often than not, to maintain a healthy weight, which leads them to seek out bariatric surgery (BS), which is the only therapy currently available for these conditions [7]. There is no doubt that surgeons and medical professionals come across these patients in their practice, as liposuction is the second most frequently carried out surgical procedure on patients in the United States between the ages of 35 and 64. The benefit of liposuction is that the removal of fat cells prevents additional fat from accumulating in those areas in the future. Liposuction ultimately gives plastic surgeons the ability to semi-permanently redistribute volume in a manner that best suits a patient, with fewer complications, morbidities, and deaths compared to other surgical techniques [8].

## Review

Obesity and cardiovascular diseases

Obesity significantly increases the risk of developing several heart conditions, including coronary artery disease, hypertension, heart failure, stroke, dyslipidemia, diabetes, and atrial fibrillation. The relationship between obesity and cardiovascular diseases is depicted below (Table 1).

**Table 1:** Relationship between obesity and cardiovascular diseases

Factor/aspect	Relationship between obesity and cardiovascular diseases
Body mass index (BMI)	Positively correlated; higher BMI increases risk.
Abdominal obesity	Strongly associated with increased cardiovascular risk.
Hypertension	Obesity is a major cause of hypertension.
Dyslipidemia	Obesity often leads to unfavorable lipid profiles.
Type 2 diabetes	Obesity is a primary risk factor for diabetes.
Atherosclerosis	Obesity accelerates the development of plaques.
Inflammation	Obesity promotes chronic inflammation.
Coronary heart disease (CHD)	Obesity substantially raises CHD risk.
Heart failure	Obesity increases the risk of heart failure.
Stroke	Obesity is linked to a higher risk of stroke.
Mortality risk	Obesity increases the risk of cardiovascular mortality.

Obesity and Coronary Artery Disease

Coronary atherosclerosis and obesity are close kinship conditions. A study on adolescent patients discovered that atherosclerosis began to develop indications of cardiovascular disease early. Patients with higher BMI values compared to those with normal body weight have more frequent and advanced atherosclerotic vascular lesions [1].** **The development of CAD, which happens when the arteries supplying blood to the heart narrow or become blocked due to atherosclerosis (plaque buildup), is strongly correlated with obesity. Angina (chest pain) and heart attacks may result from this. Longitudinal studies have shown that obesity for at least two decades is most likely a standalone coronary artery disease risk factor. Weight gain of 10 kg results in a 12% rise in the risk of coronary artery disease, and as a result, blood pressure increases by 3 and 2.3 mmHg, respectively, in the systolic and diastolic chambers [9]. Being overweight can be regarded as the most major risk factor for young people's non-ST segment elevation myocardial infarction (NSTEMI) people, preceding smoking. As BMI rises, NSTEMI incidence rises. When a person has ST-elevation myocardial infarction (STEMI), the same connection can be seen [10]. The given data shows that obesity may be connected to various vascular events in addition to age, making it a significant risk factor for STEMI. Also, BMI increases the risk of both ischemic and hemorrhagic strokes by one unit, by 4% and 6%, respectively [11].

Obesity and Hypertension (High Blood Pressure)

Extra body weight necessitates more blood to supply oxygen and nutrients to the tissues, leading to increased pressure on the artery walls. Over time, this can result in hypertension, which further increases the risk of stroke and heart disease. HF with preserved ejection fraction and HF with decreased ejection fraction, which most frequently manifests after myocardial infarction (MI), are both caused by obesity and hypertension (HTN), which are linked to metabolic syndrome and are highly prevalent in CVD patients [12]. Major factors that may lead to heart failure (HF) include obesity and blood pressure (HTN), both of which are frequently present. HTN is characterized by chronic, low-grade inflammation, which encourages unfavorable cardiac remodeling. Although macrophages are important for cardiac remodeling, an imbalance in their polarisation between the pro- and anti-inflammatory M1 and M2 phenotypes encourages excessive inflammation and cardiac damage. It has been suggested that macrophage polarisation is a result of metabolic switching between glycolysis and mitochondrial oxidative phosphorylation (OXPHOS) [12].

Pathophysiologic Mechanisms of Hypertension in Obesity

A variety of pathophysiologic mechanisms contribute to the emergence of hypertension in obese people, and these mechanisms, in turn, encourage end-organ damage like persistent kidney disorders and heart disease. Some of these intricately interconnected mechanisms comprise insulin resistance, the sympathetic nervous system, oxidative stress, adipokines, inflammation, and the renin-angiotensin-aldosterone system. Numerous of these elements, which relate to one another in bidirectional pathways, become worse by higher levels of adiposity. In general, their actions can alter the body's hemodynamics and lead to endothelial dysfunction, which encourages the rise in blood pressure that is frequently observed in obese individuals [13]. More specifically, elevated oxidative stress and decreased nitric oxide availability are both significantly linked to increased adiposity and endothelial dysfunction. Additionally, obesity is connected to increased amounts of inflammation cytokines like tumor IFN- and C-reactive protein, and necrosis factor-alpha, erythrocyte sedimentation rate along with the plasminogen activator inhibitor 1 are examples of circulating markers of inflammation [14].

The vascular effects of extra adipose tissue in obesity are a major factor in the end-organ damage linked to hypertension. Obesity is strongly associated with subclinical measures even after adjusting for other risk factors for atherosclerosis, including coronary artery calcification, increased internal and common carotid artery intimal medial thickness, and increased left ventricular mass conventional cardiovascular risk factors. Additionally, central adiposity is independently linked to an increased risk of microvascular disease and arterial stiffness, both of which significantly increase the prevalence of hypertension in this patient population, which is how the condition is mediated [13].

Obesity and Dyslipidemia

Dyslipidemia can result from obesity when the levels of cholesterol and other fats in the blood are out of balance. Cardiovascular disease risk factors include low levels of high-density lipoprotein (HDL) cholesterol (the "good" cholesterol) and high levels of triglycerides and low-density lipoprotein (LDL) cholesterol (the "bad" cholesterol). Through risk factors like elevated high levels of insulin and blood sugar, low levels of HDL cholesterol, fasting plasma triglycerides, and high blood pressure, obesity raises the risk of cardiovascular disease. Small dense LDL, postprandial hyperlipidemia with atherogenic remnant accumulation, and hepatic overproduction of apoprotein B (apoB)-containing lipoproteins are all present as new lipid-dependent, metabolic danger signs linked to weight gain [15]. Obesity is associated with higher triglycerides (TG) and free fatty acids (FFA), high-density lipoprotein-cholesterol (HDL-C) decreases with HDL dysfunction, normal or barely elevated low-density lipoprotein-cholesterol (LDL-C), and elevated small dense LDL. Additionally, apoB concentrations are frequently increased, in part due to the hepatic overproduction of apoB-containing lipoproteins.

By creating HDL particles, the liver and intestines also contribute significantly to the transport of cholesterol in reverse. HDL encourages the return of cholesterol to the liver originating from perivascular tissues, such as the arterial wall. Newly formed free cholesterol is picked up by HDL particles from nearby tissues. The lecithin-cholesterol acyltransferase (LCAT) associated with HDL then converts the cholesterol in HDL into cholesterol-esters [16]. Research into South Asians' dyslipidemia is few and far between. According to several studies, the prevalence of dyslipidemia in India as a whole varies between 10% and 73%, based on the resident's socioeconomic status, age, neighborhood, and socioeconomic strata. In urban areas, hypertriglyceridemia was more common in migrant Asians and obese Asian Indians, with a prevalence of 73% in the former group and 61% in the latter. The prevalence was somewhat lower but still higher in rural areas compared to white Caucasians. Hypertriglyceridemia was found to be 42.7% typical in urban Delhi, northern India, according to the findings of a recent cross-sectional study [17].

Obesity and Diabetes

Pre-diabetes is a condition in which a person's blood glucose levels are elevated but not high enough for diabetes to be identified. Type 2 diabetes is significantly more likely to develop in people who are obese, and it significantly raises the risk of cardiovascular diseases. Both diabetes and obesity are multifactorial, complex diseases with a high preventable mortality rate. The risk for stroke and CVD is significantly increased by both conditions. The American Heart Association has defined ideal cardiovascular health as having a fasting plasma glucose level of less than 100 mg/dL and a body mass index of less than 25 kg/m^2^ are required [18].

Due to the late-life onset, diabetes mellitus type 2 (T2DM) is also known as late-onset or adult-onset diabetes. It comprises of 95% of the total population.A person with T2DM has blood sugar levels that are higher than normal, which can exacerbate insulin resistance and deficiency [19]. The exact causes of T2DM are unknown, according to scientists, researchers, and medical experts, according to the American Diabetes Association (ADA). Several risk factors can lead to the development of T2DM. The danger signs of T2DM can either be changed or cannot be changed. High blood pressure, obesity (BMI > 30 kg/m^2^), changes in cholesterol levels, and a lack of exercise are all modifiable risk factors. Background of hyperglycemia, diabetes precursors, and/or age, race, ethnicity, family history, genetics, and gestational diabetes are the risk factors that are linked to T2DM and cannot be changed. T2DM frequently goes undiagnosed for several years, and due to the beginning of hyperglycemia being gradual, it has long-term repercussions. The body eventually starts to feel the effects, raising the risk and leading to other diseases like complications in the microvasculature. People with type 2 diabetes may have normal insulin concentrations or high insulin levels, but it is expected that their higher levels of blood sugar will result in even higher levels of insulin if their islets of Langerhans cells are functioning normally [20].

Obesity and Atrial Fibrillation

Obesity has been linked to a higher risk of atrial fibrillation (AF), a heart rhythm disorder that can lead to blood clots, stroke, and heart failure. AF is characterized by high-frequency excitation of the atrium that results in both dyssynchronous atrial contraction and irregularity of ventricular excitation. Even though AF can occur in the absence of known structural or electrophysiological abnormalities, epidemiological association studies are identifying more and more co-occurring conditions, many of which have been demonstrated to cause structural and histopathological changes that create a distinct AF substrate or atrial cardiomyopathy [21].

Several pathways may elucidate how obesity affects the development and progression of atrial fibrillation.

Inflammatory response:* *A systemic inflammatory response brought on by excess body fat releases several pro-inflammatory molecules that can harm the heart's electrical system and structure, promoting AF.

Structural changes: The left atrium may enlarge as a result of obesity, altering the structure of the heart in a way that makes it easier for abnormal heart rhythms to start and continue.

Adipokines: Adipokines are hormones that are produced by adipose tissue. An unbalanced adipokine profile in obese people may have detrimental effects on the cardiovascular system, potentially raising the risk of AF.

Recent advancements in obesity

Precision medicine, liposuction, gut microbiome studies, pharmacological interventions, bariatric surgery techniques, digital health solutions, and behavioral interventions are just a few of the recent advances in obesity research that offer hope in the fight against obesity and its harmful effects on cardiovascular health.

Pharmacotherapy

The creation of novel drugs to treat obesity is a continuous effort. Several promising medications were being tested for their potential to regulate hunger hormones, suppress appetite, and enhance metabolic functions. It's possible since the recent advancement in the development of medications, new or improved medications have been created. The Drug Enforcement Agency has currently approved five prescription drugs for ongoing treatment of obesity in adults with a BMI of 30 or 27 and comorbid conditions. Daily subcutaneous (SQ) liraglutide 3.0 mg is a notable addition to the medications authorized in 2015 [22]. Setmelanotide, a melanocortin agonist, was also authorized in 2020 for the treatment of genetic obesity syndromes affecting the proximal leptin-signaling pathway in adults and children under the age of six [23]. Despite the lack of extensive placebo-controlled studies and the presence of significant body weight effects, setmelanotide was approved because of the rarity of these syndromes.

Prescription medications approved before 2021 for non-syndromic obesity ranged from 3.4 to 8.9 kg in mean slimming down when compared to placebo over a year, with high-dose phentermine plus topiramate producing the biggest weight loss. All medications show a higher percentage of responders losing five percent of initial weight than with placebo, with orlistat having an odds ratio (OR) of 2.70 and phentermine/topiramate having an OR of 9.22 [23]. Despite being approved for short-term use, phentermine monotherapy is still the most frequently prescribed anti-obesity drug and is frequently used off-label for long-term treatment. An indication of the potential of precision therapies that target underlying mechanisms is provided by the approval of setmelanotide for the treatment of some uncommon monogenic obesity in children and adults. Drugs for long-term weight management have been mentioned in Table 2.

**Table 2:** Drugs for long-term weight management GLP-1 - glucagon-like peptide 1 Sources: [22,23]

Drug name	Purpose	Precautions
Orlistat	Fat absorption inhibitor	Gastrointestinal side-effects such as diarrhea and oily stools.
Phentermine-topiramate	Appetite-suppressant	Potential for increased rate and blood pressure. It should not be used by individuals with certain heart conditions or a history of substance abuse.
Buproprion-naltrexone	Appetite-suppressant	May increase the risk of seizures. Not recommended for individuals with a history of eating disorders or opioid use disorder.
Liraglutide	GLP-1 receptor agonist	May cause nausea and vomiting. Caution in individuals with a history of pancreatitis.
Semaglutide	GLP-1 receptor agonist	Potential for gastrointestinal side-effects nausea and diarrhea.
Naltrexone-buproprion	Appetite-suppressant	May increase the risk of suicidal thoughts and behaviors in some individuals.

Gut Microbiome

The relationship between the gut microbiome and obesity is still a hot topic for research. Researchers are examining how gut bacteria affect metabolism and weight gain, and they are working to create specialized medications that target the microbiome. Genetic, behavioral, socioeconomic, and environmental factors all have an impact on obesity, making it a complex and multifactorial disease. Studies on mice and the effect of gut microbiota on body fat accumulation have shown that gut bacteria play a significant role in obesity. It has been acknowledged that the gut microbiome, which is primarily made up of bacteria, is an important aspect of human health. It is crucial to define specific terms because the terminology used in this field can be difficult to understand. The term "microbiota" describes the diversity of microorganisms, primarily bacteria, in a specific environment. Metagenome refers to the genetic makeup of the microbiota. Due to the vast amount of research, this paper concentrates on a few key issues and illustrative studies about the connection between gut microbes and obesity [24].

The application of molecular techniques based on 16S ribosomal RNA (rRNA) genes independent of culture in the 1990s, such as restrictions fragment length polymorphisms, gel electrophoresis using temperature-gradient denaturing, and fluorescent hybridization in situ, competitive PCR, and PCR in real-time, increased our understanding of the Bacteroides and Firmicutes, which predominate in the human stomach microbes [24].

When lean and obese people are compared, there is weight increase and larger levels of Firmicutes and lower levels of Bacteroidetes in the gut microbiota.** **The ratios of these bacterial groups changed after gastric bypass and diet therapy. In comparison to people with a normal BMI and anorexic patients, obese subjects also displayed a lower proportion of Bacteroidetes, but no discernible variation in Firmicutes abundance was found [24]. Another study comparing obese and lean twins discovered that while there was no discernible difference in the proportion of Firmicutes, obese individuals had lower bacterial diversity and a higher abundance of Actinobacteria [25]. The F/B ratio, or the abundance ratio of Firmicutes to Bacteroidetes, was first proposed as a potential biomarker for susceptibility to obesity. The connection between the intestinal microbiome and obesity could be more complex than just a simple imbalance of these bacterial groups, according to further research and meta-analyses of data on the gut microbiome and obesity status [25].

Gut Microbiota Alteration by Diet and Antibiotics

It's crucial to verify how diet affects gut microbiota, given the common knowledge that gut microbes depend on food consumed by hosts. Mice were fed a high-fat diet both freely (without resistin-like molecule) and with resistin-like molecule (RELM) in a comprehensive investigation to compare gut microbiota of the wild-type and RELM defective strains. Because obesity caused by a high-fat diet cannot affect a mouse lacking the RELM. Results revealed that both groups of mice experienced similar changes in gut microbial composition, even though RELM-deficient mice remained relatively lean, indicating that the effects of diet have a greater impact on gut microbiota than the phenotype of obesity [25]. Attention has also been paid to the enduring effects of antibiotics on stomach flora, given how frequently bacteria are treated with antibiotics. Clindamycin treatment for seven days in humans resulted in long-term changes to the Bacteroides population with no signs of recovery for up to two years after administration. Another discovery demonstrated a significant reduction in the diversity of bacteria in the human gut following brief exposure to the antibiotic ciprofloxacin. Within four weeks of the intervention, the microbiota mostly returned to its pretreatment state, but by six months, several taxa didn't seem to have recovered [24].

The gut microbiota stands out as a relatively brand-new risk factor with a much more intimate impact when compared to well-known risk factors like diet, lifestyle, and socioeconomic status. Although attempts to summarise the complex interactions between gut microbiota and obesity by compositional data continue to be made, it is undeniably true that what we eat has an impact on our gut microbiota, which in turn affects inflammation and host metabolism. Based on a recent study, prebiotics helped overweight and obese children lose weight, reduce fat deposition, and possess greater levels of Bifidobacterium spp. and reduced amounts of Bacteroides vulgatus in their serum [25]. Therefore, altering gut flora through dietary means and dietary medications is not just a potentially effective way to treat obesity but also a theoretically effective method of reducing its symptoms.

Bariatric Surgery Techniques

With a focus on minimally invasive procedures and better patient outcomes, improvements in surgical techniques for the treatment of obesity were being investigated. In terms of long-term weight loss and the remission of metabolic diseases like type 2 diabetes, bariatric surgery has produced encouraging results. Modern surgical techniques have lowered risks and enhanced results, making these procedures more affordable for qualified patients. Patients who are extremely obese find it difficult to lose weight and, more often than not, to maintain a healthy weight. As a result, they turn to bariatric surgery (BS), which is currently the only effective treatment for these patients. After BS, there is a significant change in the diversity and structure of gut microbiota (GM). Additionally, it is now widely acknowledged that micronutrient deficiencies can cause severe deficiency-related disorders, the most common of which are anemia (10-74%) and neurological dysfunctions (5-9%) [6]. The fundamental idea behind bariatric surgery serves as the recognition that a disease with severe obesity has numerous adverse health effects that, in patients who have not been overweight, can be improved or reversed, able to maintain non-surgical methods for losing weight. An NIH consensus panel developed the standards for surgical intervention in 1991 [26]. Both temporary and permanent weight loss may be accelerated by pharmacotherapy; bariatric surgery is appropriate for everyone with a BMI (kg/m^2^) >40 and for people with a BMI of 35 to 40 who also have comorbid conditions, according to specific guidelines established by the NIH consensus panel. Specific standards for bariatric/metabolic surgical intervention in patients with less severe cases of obesity, like those with a BMI of 30-35 and type 2 diabetes, have been established, but they continue to be valid today after 24 years. Bariatric surgery eligibility requirements are quickly evolving to consider both how serious obesity is as determined by BMI and the presence or absence of comorbid conditions [27].

The appropriate surgical risk must be evaluated for candidates for bariatric surgery, which includes having a history of respiratory, cardiovascular, and other system diseases, as well as how these concurrent illnesses are managed. These guidelines apply to all surgical procedures. For instance, it is conceivable that individuals with a very high risk of developing end-effects of cardiovascular disease will have occurred at that point to an elevated perioperative risk and that it is unlikely to be successful in reversing cardiovascular disease by enhancing the risk profile. Patients, however, who experience enlarged heart failure, breathing difficulty, or anasarca and immobility are a few instances among those who suffer from extreme obesity in whom weight loss lowered perioperative risk [26].

Particular Bariatric Surgical Techniques

At one time, it was believed that surgical procedures could have three different outcomes: restrictive, malabsorptive, or a combination of the two. It is now clear that this concept is incomplete and, in some ways, oversimplified. Numerous pieces of evidence point to the existence of numerous neural and endocrine signaling pathways that regulate energy intake, eating behaviors, appetite suppression, satiety, and possibly physical activity.

Gastric Bypass Roux-en-Y

Mason created the gastric bypass Roux-en-Y in the 1970s in response to the ileojejunal intestinal bypass's unacceptable complication rates, a procedure that caused malabsorption, reduced appetite, and significant weight had benefits, but the complication rates were too high [28]. To create a digestive pouch with a one-ounce capacity, the stomach is split in half during this procedure. To redirect the nutrients to remove the proximal jejunum, duodenum, and stomach, a Roux-en-Y gastrojejunostomy is performed. The vagal trunks are left undisturbed, but during the procedure, several different branches leading to the stomach body are split apart.

Sleeve Gastroplasties

According to the stomach's more pronounced curvature, a tubular stomach is created during this procedure by resecting about 80% of the body of the stomach. There isn't a need for an anastomosis from the stomach to the small intestine. Even though there may be certain dietary restrictions, abdominal emptying is accelerated [26].​​​​​​

Surgical Implant of Devices

The proximal stomach is wrapped in an adjustable gastric band to reduce the size of the gastric pouch and outlet. A balloon attached to a subcutaneous port can change the gastric emptying rate. This method creates an intermittent vagal blockade by wrapping leads tightly around vagal branches at the diaphragm. Weight loss occurs as a result of reducing appetite and establishing early satiety. It is believed that intermittent blockade prevents neural adaptation, as it did in the case of truncal vagotomy. A tool for this use has received approval from the Food and Drug Administration [29]. While several endoscopically placed or sutured devices are still being developed, the Food and Drug Administration recently approved the use of gastric balloons.

Duodenal Switch and Biliopancreatic Diversion

A sleeve gastrectomy is performed in this more complicated procedure. It is possible to achieve an anastomosis between the proximal duodenum and the bypassed intestine, which results in some degree of nutrient malabsorption. As a result of the greater potential for both immediate and long-term complications, this procedure is rarely carried out [26].

Safety of Bariatric Surgery

The advantages of weight loss for those with severe obesity, especially those who also have comorbid ailments, cannot be overstated, but these benefits must be weighed against possible surgical risks. Up to ten times more frequently than they do now, complications like perioperative mortality were common in the past. In Flum and Dellinger's population-based research, for instance, 2% of people die after a gastric bypass, and this is significantly higher than the 0.5% typically disclosed by surgeons who choose to disclose their results [30].

Liposuction

Liposuction is mostly an artistic procedure rather than a medicinal one. It necessitates the accurate and proficient implementation of scientific knowledge in real-world scenarios, and it is a skill learned through practical experience in a clinical setting. The patient experiences the same level of satisfaction as the surgeon while both engage in the challenging process of attaining the desired goal. Similar to how the ocular lens is phacoemulsified to treat cataracts, liposuction removes fat. Localized fat deposits can be removed using tiny incisions that leave a barely noticeable scar. A buildup of fat in the glute-crural areas, abdominal region, and hips are the main warning signs. While a trim, athletic body shape is ideal, lipo-sculpture is expected to achieve the desired chest and shoulder contours and flattened thigh and hip areas that are both narrow. Age and race also affect the patterns of localized fat accumulation. With age, there is an increase in the amount of fat inside the abdomen and a decrease in the subcutaneous fatty layer. In comparison to men, women had a fat synthesis pattern and a proportionately higher body fat percentage than men, known as the gynaecoid pattern, which is characterized by a rise in deposits in the lateral buttock and thigh, hip, and truncal area. In contrast, men have a pattern for the android, which focuses on the abdominal and truncal areas [31]. Because unevenly distributed fat cells are permanently removed during liposuction, it is effective at changing contour.

Cosmetic Indications

Liposuction is used to remove extra fat deposits from troublesome body parts to achieve body contouring. Fat is suctioned out of defined body parts that can be contoured. The lumbar region, gluteal region, trochanteric region, abdomen, and flanks are the areas most frequently treated for fat amid the gluteal fold and inframammary fold. The thighs, calves, and breasts (such as through breast reduction surgery) are additional areas where fat can be removed. The surgeon should select areas where wide cannula fanning is possible during the procedure and where clothing can conceal the surgical scar, which is modest because the location of the incision is crucial in terms of anatomy. The inferolateral iliotibial tract, distal posterior thigh, mid-medial thigh, gluteal crease, and lateral gluteal depression are the locations where superficial subcutaneous tissues attach to the underlying deep muscle fascia. The body's natural shape is defined by these regions, so suctioning from them increases the likelihood of contour irregularities. Ideal conditions for patients to achieve the desired aesthetic results include having enough skin elasticity and weighing between 20% and 30% of one's ideal weight [8]. For post-surgical bariatric patients, liposuction is being used more frequently as a complement to enhance other aesthetic procedures like gluteal fat transfer, body contouring, breast augmentation, cervicoplasty, and abdominoplasty.

Non-cosmetic Indications

Additionally, liposuction is utilized for restoration purposes, including the following ailments' treatment. Angiolipomas and lipomas leave little to no scarring. Lymphedema, particularly when it is unresponsive to traditional treatment approaches. The occurrence of fat loss in the neck and upper back region is associated with the use of HIV medications and the presence of Cushing syndrome. The procedure involves combining mammoplasty with the treatment of male gynecomastia and female macromastia.

Variations of Liposuction

The ablative tool ultrasound is used in urology and neurosurgery. Ultrasonic-assisted liposuction (UAL) was developed and made popular in Italy by Zocchi in the early 1990s [31]. His original purpose for using ultrasound to aspirate fat was to extract collagen. The accidental finding that adipose tissues were successfully emulsified with in vitro preservation of connective tissue structures gave rise to the idea of employing ultrasound as an adjunct in person.

Other surgical specialties have discontinued the use of internal ultrasound in ultrasonic liposuction due to its perceived limited advantages, elevated risk of skin burns and seroma development, and lack of further benefits compared to conventional liposuction. Ultrasonic liposuction has received support from the American Society of Plastic and Reconstructive Surgeons. In the West, where the procedure is frequently used in large numbers, tumescent liposuctions with a large volume have become more popular [31]. Patients who are carefully chosen, have reasonable expectations and are aware of the procedure's restrictions can have liposuction safely performed on them. Typically, they are incredibly pleased with the outcomes.

Behavioral Intervention and Digital Health

As obesity has psychological and emotional components, researchers were looking at behavioral interventions. Programs to manage obesity were incorporating cognitive-behavioral therapy as well as other methods. To assist people in successfully managing their weight, mobile apps, wearable technology, and other digital health solutions were being developed. These innovations sought to increase adherence to healthy practices, track advancement, and offer tailored recommendations.

## Conclusions

Cardiovascular disease and obesity together pose a serious health threat in the modern world. It's essential to comprehend the underlying mechanisms and risk factors linked to obesity-related CVD in order to create efficient preventive and therapeutic measures. Precision medicine, gut microbiome studies, pharmacological interventions, and behavioral interventions, among other recent developments in obesity research, offer hope in the fight against obesity and its harmful effects on cardiovascular health. We can make significant progress in lowering the prevalence of obesity and enhancing the general health and well-being of people all over the world as we continue to learn more about it.
